# Nineteen-Month Immunity to Adverse Radiation Effects Following 5-Fraction Re-radiosurgery With 43.6 Gy for Local Progression After Prior 3-Fraction Radiosurgery for Brain Metastasis From Pan-Negative Lung Adenocarcinoma

**DOI:** 10.7759/cureus.46374

**Published:** 2023-10-02

**Authors:** Kazuhiro Ohtakara, Makoto Nakao, Hideki Muramatsu, Kojiro Suzuki

**Affiliations:** 1 Department of Radiation Oncology, Kainan Hospital Aichi Prefectural Welfare Federation of Agricultural Cooperatives, Yatomi, JPN; 2 Department of Radiology, Aichi Medical University, Nagakute, JPN; 3 Department of Respiratory Medicine, Kainan Hospital Aichi Prefectural Welfare Federation of Agricultural Cooperatives, Yatomi, JPN

**Keywords:** carcinoembryonic antigen, cyfra 21-1, hypofractionation, bullous pemphigoid, pan-negative, lung adenocarcinoma, re-irradiation, biologically effective dose, stereotactic radiosurgery, brain metastasis

## Abstract

Clinical management of patients with local control failure following stereotactic radiosurgery (SRS) for brain metastasis (BM) can be frequently challenging. Re-irradiation with multi-fraction (fr) SRS by using a biological effective dose of ≥80 Gy, based on the linear-quadratic formula with an alpha/beta ratio of 10 (BED_10_), can be an efficacious option for such a scenario with the BED_10_ of <80 Gy. However, its long-term safety beyond one year remains unclear. In this report, we describe the case of a patient with a single metachronous BM from lung adenocarcinoma (LAC), without major genetic alterations, in which re-SRS with 43.6 Gy/5 fr (BED_10_ 81.6 Gy) for local progression, following prior 3-fr SRS of the BM, resulted in sustained regression without any local adverse radiation effects (AREs) for 19 months.

The BM with a gross tumor volume (GTV) of 1.12 cm^3^ in the left parietal lobe was initially treated with SRS of 27 Gy/3 fr (50% isodose). Despite steroid administration for nivolumab-induced bullous pemphigoid associated with transient elevation of tumor markers, the BM showed local progression with T1/T2 matching at 38.3 and eight months after SRS and discontinuation of nivolumab, respectively. In the 5-fr re-SRS, 99% of the GTV (1.18 cm^3^) was covered with 43.6 Gy (63% isodose). However, along with the thoracic disease progression, multiple new BMs developed 15.5 months after the re-SRS, for which volumetric-modulated arc-based whole brain radiotherapy (WBRT) was administered, with simultaneously integrated boosts to 17 lesions and moderate dose attenuation in the pre-irradiated region. However, concurrent administration of gemcitabine and WBRT might have led to persistent severe anorexia for 2.5 months. The patient died 10.8 years after the initial chemotherapy. The relatively small GTV with the superficial location may have rendered the re-irradiated region immune to AREs after the high BED_10_ re-SRS. Long-term survival can be achieved by chemoimmunotherapy in patients with pan-negative LAC, with limited systemic metastases who are unfit for targeted agents. Therefore, SRS for limited BMs in such scenarios should aim for complete local tumor eradication beyond a partial response in either a first-line or re-irradiation setting.

## Introduction

Clinical management of patients harboring local progression following stereotactic radiosurgery (SRS) for brain metastasis (BM) is frequently challenging, especially for single- or oligo-BMs, refractory to systemic therapy, without any extra-central nervous system (CNS) active disease (isolated CNS failure) [1,2]. Determining whether tumor regrowth (true progression), radiation injury, or transient enlargement of a degenerated tumor (pseudo-progression) predominates is frequently difficult, and the diagnostic criteria remain controversial and unclear [1-3]. Re-irradiation with single- or multi-fraction (fr) SRS (re-SRS) is one of the available treatment options for such scenarios that are deemed to have a dominance of viable tumor tissue; the target definition, marginal dose fractionation, and the dose gradient outside and inside the lesion boundary vary substantially between facilities, and the optimal scheme remains undetermined [3,4]. A non-curative and conservative dose is commonly administered for re-SRS, given the risk of radiation injury not amenable to medical management [1,2]. In addition, a relatively homogeneous target dose is frequently used, especially in considerable linac-based SRS, regardless of whether it is the initial treatment or a re-treatment [5]. Persistent viable tissue after failed SRS is likely associated with a poorly demarcated brain-tumor interface, with a high predisposition to profound invasion into the surrounding parenchyma, leading to therapeutic conundrums [6].

To improve the insufficient long-term efficacy and safety of general SRS for BMs, dose escalation to the boundary and interior of the gross tumor volume (GTV) has been performed in first-line and re-irradiation settings since 2018: a GTV margin defined by T1/T2 matching is the basis of the dose prescription and planning, and the GTV is sufficiently covered with a biological effective dose of ≥80 Gy, based on the linear-quadratic formula with an alpha/beta ratio of 10 (BED_10_), along with concentrically laminated steep dose increase inside the GTV boundary (extremely inhomogeneous GTV dose) and a moderate dose spillage margin outside the GTV [3,4,7,8]. Re-SRS with the above scheme and a flexible and adequate dose fractionation can yield a superior tumor response with minimal adverse radiation effects (AREs) for selected cases with local failure following prior SRS with the BED_10_ of <80 Gy; however, its long-term safety and durability beyond one year remains unclear [3,4].

In this report, we discuss a case of a single metachronous BM from lung adenocarcinoma (LAC), without major genetic alterations, where re-SRS with 43.6 Gy/5 fr for local progression, following prior SRS with 27 Gy/3 fr for the BM, resulted in sustained regression without any local AREs for 19 months.

This report was part of the clinical study approved by the Clinical Research Review Board of Kainan Hospital Aichi Prefectural Welfare Federation of Agricultural Cooperatives (20220727-1).

## Case presentation

**Figure 1:**
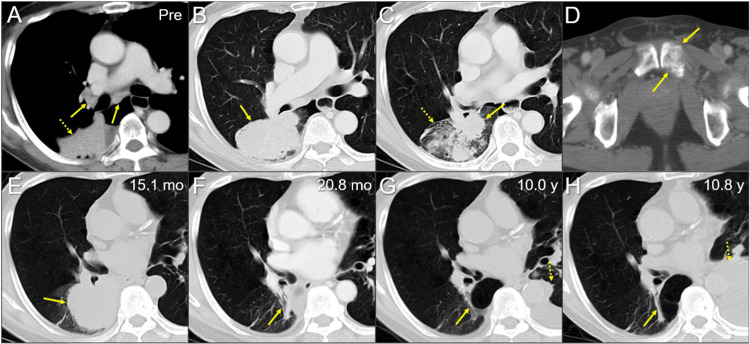
Contrast-enhanced (CE) and non-CE thoracic and pelvic CT images The images show axial contrast-enhanced (CE) CT images (A-D, F); non-CE CT images (E, G, H); thorax (A-C, E-H); pelvis (D); soft tissue window (A); lung window (B, C, E-H); bone window (D); before first-line chemotherapy (Pre) (A-D); at 15.1 months (mo) after the first-line chemotherapy (E); at 20.8 months (F); at 10.0 years (y) (G); and at 10.8 years (H) (A-H) These images are shown at the same magnification and coordinates under co-registration and fusions. (A-C) Multiple lymph node swelling (arrows in A) in the right hilar and subcarinal regions and a well-demarcated mass lesion (arrows in B, C) associated with secondary changes due to partial atelectasis (dashed arrow in A) and pneumonitis (dashed arrow in C) in the right lower lobe. (D) An osteolytic lesion with a pathological fracture in the left pubic bone (arrows in D). (E) At 15.1 months, the perihilar lesion (arrow in E) was refractory to fourth-line chemotherapy. (F) At 20.8 months (after six courses of fifth-line chemotherapy), the lesion regressed remarkably, only leaving scar tissue (arrow in F). (G, H) At 10.0 to 10.8 years, the lesion remained in complete regression (arrows in G, H), while pleural dissemination (dashed arrows in G, H) progressed in the contralateral thorax CT: computed tomography

A 66-year-old right-handed male, an ex-smoker, presented with hemosputum, for which repeated sputum cytological examinations were negative; thoracic plain radiography performed nine months earlier showed no obvious abnormality. Three months later, the patient developed right shoulder pain and was noted to have a right hilar mass (Figures 1A-1C).

**Figure 2:**
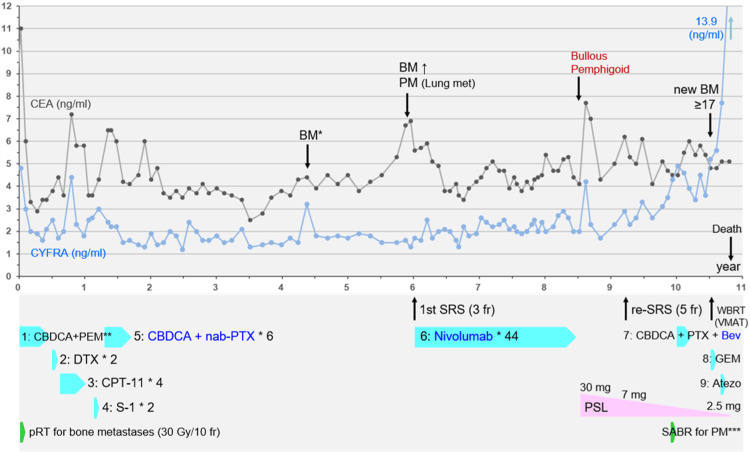
Summary of anti-cancer treatments along with a series of changes in the carcinoembryonic antigen and cytokeratin 19 fragment antigen 21-1 levels *A tiny BM was retrospectively identified in this review. **CBDCA+PEM: four courses of CBDCA plus PEM followed by two courses of maintenance PEM. ***At 9.0 years, a persistent and progressive solitary lung metastasis in the left upper lobe was treated with SABR. CEA: carcinoembryonic antigen; CYFRA: cytokeratin 19 fragment antigen 21-1; BM: brain metastasis; PM: pulmonary metastasis; met: metastasis; BP: bullous pemphigoid; SRS: stereotactic radiosurgery; fr: fraction; WBRT: whole brain radiotherapy; VMAT: volumetric-modulated arc therapy; CBDCA: carboplatin; PEM: pemetrexed; DTX: docetaxel; CPT-11: irinotecan; nab-PTX: nanoparticle albumin-bound paclitaxel; PTX: paclitaxel; Bev: bevacizumab; GEM: gemcitabine; Atezo: atezolizumab; PSL: prednisolone; pRT: palliative radiotherapy; SABR: stereotactic ablative radiotherapy

The Karnofsky performance status (KPS) was 90%. The initial clinical stage, based on ^18^F-fluorodeoxyglucose positron emission tomography/CT, was IV B (cT3N2M1c), based on the tumor, node, and metastasis (TNM) grading system defined by the Eight Union for International Cancer Control criteria. The endobronchial biopsy showed LAC without major genetic alterations that could be examined at the time. The patient's past medical history included acute myocardial infarction, paroxysmal atrial fibrillation, and chronic obstructive pulmonary disease. The anti-cancer treatments are summarized in Figure 2, which were initiated nearly five months after the onset of relevant symptoms.

**Figure 3:**
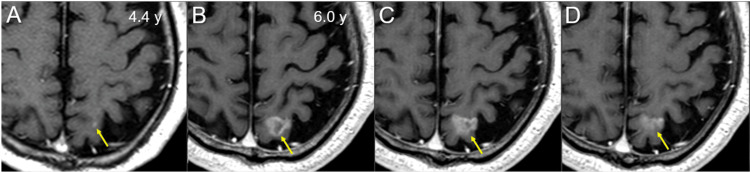
MRI findings at the diagnosis of the brain metastasis The images show CE axial T1-weighted images (WIs); at 4.4 years (y) after the initiation of the first-line chemotherapy; and at 6.0 years (B-D) (A-D) These images are shown at the same magnification and coordinates under co-registration and fusions (A, B: almost the same cross-section; C, D: different cross-sections). (A) A tiny enhancing lesion in the left superior parietal lobule was retrospectively identified. (B-D) A heterogeneously enhancing irregularly-shaped lesion located mainly in the cerebral cortex, with infiltration to the brain surface MRI: magnetic resonance imaging; CE: contrast-enhanced

The patient was initially treated with first-line chemotherapy and conventional external-beam radiotherapy with 30 Gy/10 fr for two painful bone metastases in the right scapula and the left pubis (Figure 1D), which were the only distant metastases (Figure 2). The first- to fourth-line chemotherapy resulted in short-term progression (Figure 1E); the fifth-line chemotherapy with six courses of carboplatin plus nanoparticle albumin-bound paclitaxel (nab-PTX) resulted in near-complete remission (Figure 1F). The patient was followed up thereafter. Although there was a progression-free period for more than four years, the patient was then diagnosed with an asymptomatic single BM (Figure 3) and a single pulmonary metastasis (PM) in the left upper lobe (images not shown), along with the elevation of carcinoembryonic antigen (CEA) levels only, six years after the initiation of the first-line chemotherapy (Figure 2).

**Figure 4:**
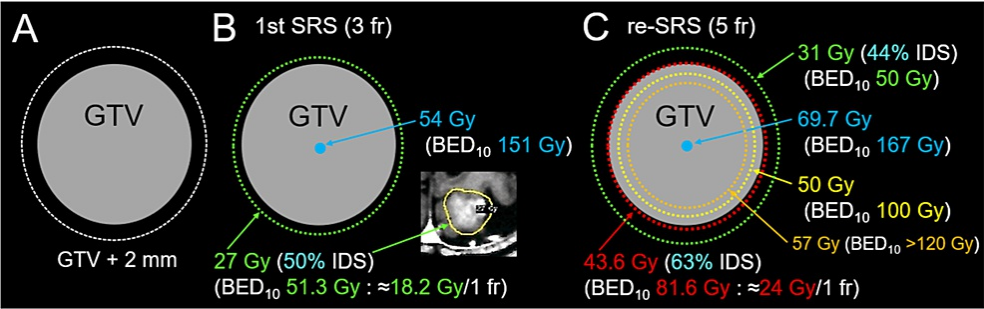
The target definition for evaluation and differences in planning schemes of the initial and salvage stereotactic radiosurgery (A) The GTV + 2 mm structure is generated by adding an isotropic 2 mm margin to a GTV. (B) The prescription isodose surface (IDS) of 27 Gy intentionally encompasses approximately 0.5-1 mm outside the GTV (1.12 cm^3^) boundary, where the GTV marginal dose, e.g., D_98%_, is unknown. The inset shows the 50% isodose line superimposed onto a CE-T1-WI. (C) In re-SRS, 99% of the GTV (1.18 cm^3^) is covered with 63% IDS of 43.6 Gy (BED_10_ 81.6 Gy, equivalent to a single dose of 24 Gy). The dose gradient inside and outside the GTV boundary is characterized by concentrically laminated steep dose increase inside the GTV from 81.6 Gy to 120 Gy as a BED_10_ and the appropriate dose spillage margin with the BED_10_ of 50 Gy covering 2 mm outside the GTV, respectively, which can be achieved by intentionally allowing high doses inside the GTV without any dose constraints SRS: stereotactic radiosurgery; fr: fraction; GTV: gross tumor volume; GTV + 2 mm: a structure generated by adding an isotropic 2 mm margin to the GTV boundary; D_98%_: a minimum dose covering at least 98% of the target volume; BED_10_: a biologically effective dose based on the linear-quadratic formula with an alpha/beta ratio of 10; IDS: isodose surface (% IDS normalized to 100% at the maximum dose); CE: contrast-enhanced; WI: weighted image

The BM was retrospectively identifiable as a tiny lesion at 4.4 years (18.7 months before the diagnosis) (Figure 3A). The BM (1.12 cm^3^) was treated with 3-fr SRS on three consecutive days using Leksell Gamma Knife (LGK) (Elekta AB, Stockholm, Sweden) at another facility, in which the 50% isodose surface (IDS) with 27 Gy encompassed the enhancing lesion sufficiently (Figure 4B).

**Figure 5:**
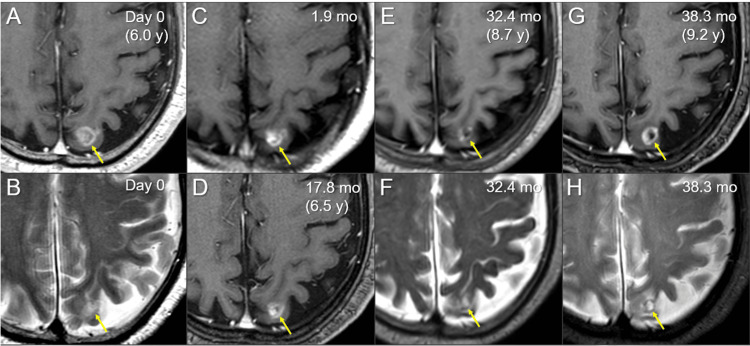
MRI findings before and after the initial stereotactic radiosurgery The images show axial CE-T1-WIs (A, C, D, E, G) and axial T2-WIs (EB, F, H); on the day (Day 0) of SRS (6.0 years [y] after the first-line chemotherapy) (A, B); at 1.9 months (mo) after SRS (C); at 17.8 months (D); at 32.4 months (E, F); and at 38.3 months (9.2 years after the first-line chemotherapy) (G, H) (A-H) These images are shown at the same magnification and coordinates under co-registration and fusions. Therefore, some images with thick slices are slightly blurry (B, C, E, F, H). (C, D) T2-WIs were unavailable at 1.9 and 17.8 months. (A-F) The lesion regressed gradually (arrows in A-D), leading to the nadir response (arrows in E, F) with minimal enhancing effects and surrounding edema. (G, H) At 38.3 months, the enhancing effects (arrow in G) increased significantly, and the configuration of the mass visible on T2-WIs (T2-mass, arrow in H) became clearer MRI: magnetic resonance imaging; CE: contrast-enhanced; WIs: weighted images; SRS: stereotactic radiosurgery

Nivolumab as the sixth line was administered thereafter. Although there was a progression-free period for more than two years, nivolumab was discontinued after 44 courses (29.8 months after the initiation) due to the development of bullous pemphigoid (BP) that was deemed an immune-mediated adverse event, for which steroids were effectively administered and thereafter tapered (Figure 2). A transient increase in both CEA and cytokeratin 19 fragment antigen 21-1 (CYFRA) was observed after steroid introduction (Figure 2). The BM remained regressed 32.4 months after SRS; however, local progression was observed six months later (eight months after the discontinuation of nivolumab), despite continued steroid administration (Figures 2, 5).

**Figure 6:**
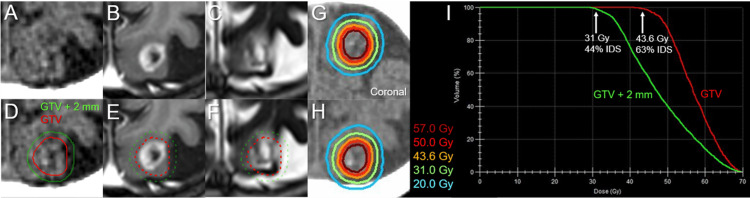
Target definition, planned dose distribution, and dose-volume histograms for the re-radiosurgery The images show CT findings (A, D, G, H); CE-T1-WIs (B, E); T2-WIs (C, F); target definitions (D, E, F); dose distributions (G, H); axial views (A-F, H); a coronal view (G); and dose-volume histograms (I) (A-F) These images are shown at the same magnification and coordinates under co-registration and fusions. (D-F) The contours of the defined GTV and the GTV + 2 mm structure are superimposed onto each image, where the GTV is intentionally oversized to include the surrounding brain surface. (G, H) Five representative isodose lines are superimposed onto CT images. (I) The arrows indicate the coverage of the GTV and GTV + 2 mm with 43.6 Gy and 31.0 Gy, respectively GTV: gross tumor volume; IDS: isodose surface; CT: computed tomography; CE: contrast-enhanced; WIs: weighted images

The local progression was deemed to have dominance of viable tumor regrowth. Therefore, the sole active lesion was re-treated with 5-fr SRS with a sufficient BED_10_, an internal steep dose increase, and a moderate dose spillage margin outside the GTV as described in Figures 4C, 6, and Table 1.

**Table 1:** Planning parameters for the 5-fraction re-radiosurgery *The BED_10_s for absolute doses of 43.6 Gy and 31.0 Gy in 5 fractions are 81.6 Gy and 50.2 Gy, respectively. **The X Gy volume (vol.) is the irradiated isodose volume receiving at least X Gy, including the GTV GTV: gross tumor volume; D_max_: maximum dose; BED_10_: a biologically effective dose based on the linear-quadratic formula with an alpha/beta ratio of 10; D_98%_: a minimum dose encompassing at least 98% of the target volume; D_min_: minimum dose

Structures	Parameters	Dosimetric goals	Parameters
GTV	Volume		1.18 cm^3^
	D_max_ (BED_10_)	≥56.4 Gy (120 Gy)	69.7 Gy (166.9 Gy)
	D_98%_ (BED_10_)	≥43 Gy (80 Gy)	45.0 Gy (85.5 Gy)
	43.6 Gy coverage*		99.0%
	D_min_		40.1 Gy (72.3 Gy)
GTV – 2 mm	D_98%_ (BED_10_)	≥50 Gy (100 Gy)	57.0 Gy (122.0 Gy)
GTV + 2 mm	Volume		2.68 cm^3^
	D_98%_ (BED_10_)		32.1 Gy (52.7 Gy)
	31.0 Gy coverage*	≥95%	99.1%
Body	43.6 Gy vol.		1.66 cm^3^
	24.0 Gy vol.	<20 cm^3^	5.13 cm^3^

The re-SRS was implemented using a multileaf collimator Agility® (Elekta AB) mounted in a linac Infinity® (Elekta AB) with a 6 megavoltage (MV) X-ray flattening filter (FF)-free beam [4,8]. The planning system employed was Monaco® (Elekta AB), and the dose calculation algorithm was an X-ray voxel Monte Carlo. The dose distribution was optimized with volumetric-modulated arcs (VMA). The dedicated software MIM Maestro^TM^ (MIM Software, Cleveland, OH) was used for image co-registration, fusion, and contouring. Each delivery was performed under image guidance based on an XVI cone-beam CT (Elekta AB), and a HexaPOD six degrees of freedom robotic couch (Elekta AB).

**Figure 7:**
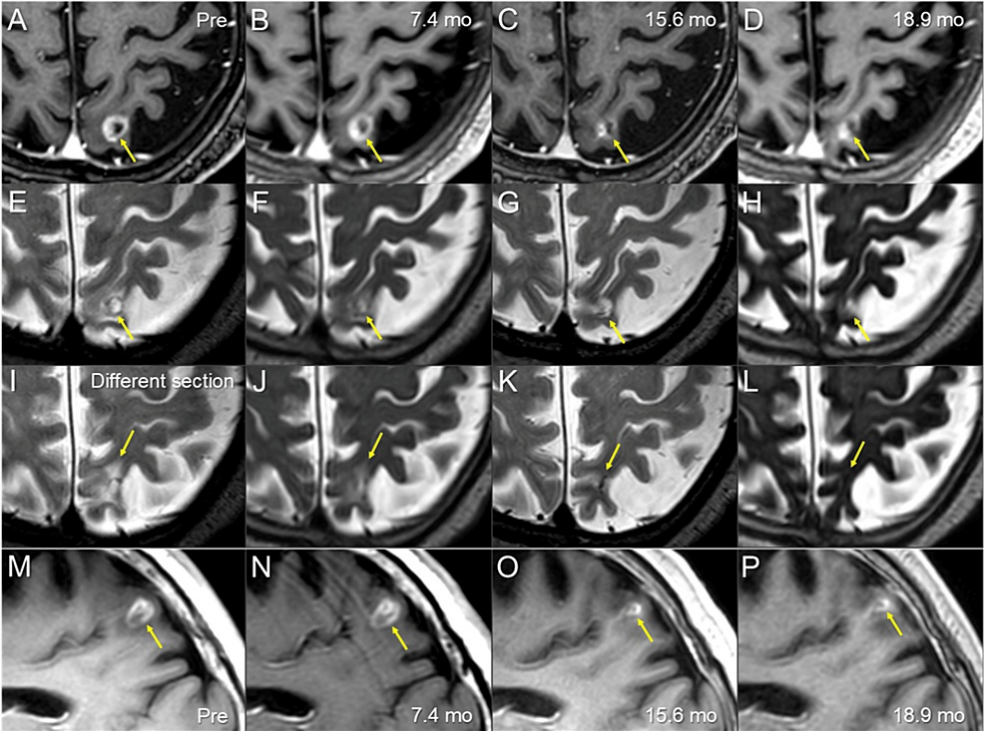
MRI findings before and after the re-radiosurgery for the local progression following prior radiosurgery The images show CE-T1-WIs (A-D, M-P); T2-WIs (E-L); axial images (A-L); sagittal images (M-P); before re-SRS (Pre) (A, E, I, M); at 7.4 months (mo) after the re-SRS initiation (B, F, J, N); at 15.6 months (C, G, K, O); and at 18.9 months (D, H, L, P) (A-P) These images are shown at the same magnification and coordinates (A-H, M-P) under co-registration and fusions; the section of the images I-L is different from that of the A-H. (A, E, I, M) The heterogeneously-enhancing lesion (arrows in A, M) and the corresponding ring-like lesion visible on the T2-WI (T2-mass, arrow in E), a T1/T2 match, are associated with perilesional edema (arrow in I). (B, F, J, N) At 7.4 months, the enhancing lesion (arrows in B, N) and the T2-mass remained almost unchanged; the surrounding high intensity became slightly less noticeable (arrow in J). (C, G, K, O) At 15.6 months, the enhancing effects and the lesion volume decreased significantly (arrows in C, O), the T2 mass became slightly obscured and some became low-intensity scar-like structures (arrows in G, K). The surrounding edema almost disappeared (arrows in G, K). (D, H, L, P) At 18.9 months, the enhancing effects became slightly more noticeable (arrows in D, P); the T2 mass became more obscure and the surrounding edema continued to disappear (arrows in H, L) MRI: magnetic resonance imaging; CE: contrast-enhanced; WIs: weighted images; SRS: stereotactic radiosurgery; T2 mass: a visible mass on T2-WIs

The enhancing effects and the lesion size gradually decreased, with the visible mass on T2-weighted images also becoming obscured, and leaving a scar-like structure, along with attenuation of the surrounding edema (Figure 7).

**Figure 8:**
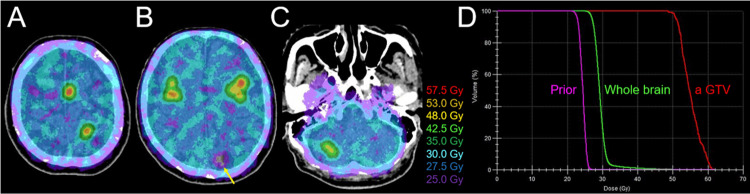
Dose distributions and dose-volume histograms for VMA-based WBRT with simultaneously integrated boosts to 17 lesions The images show dose distributions (A, B, C); and DVHs (D) (A-C) The representative isofill doses are superimposed onto non-CE CT images. The dose in the previously irradiated region was intentionally moderately reduced to control potential dissemination and to reduce the risk of radiation injury (arrow in B). The doses to the bilateral auditory apparatuses are significantly reduced, compared with those for conventional opposed-field WBRT. (D) The DVHs of the largest GTV (0.33 cm^3^) in the left frontal lobe, the whole brain, and the prior irradiated region (Prior) are representatively shown. The D_98%_ and D_50%_ of the GTV are 50.5 Gy and 54.9 Gy, where sufficient GTV coverage with 53 Gy (BED_10_ >80 Gy) is intentionally withheld to reduce the risk of radiation injury. The D_98%_ and D_50%_ of the whole brain are 26.6 Gy and 29.2 Gy, and 30 Gy encompasses 28% of the whole brain. The D_98%_, D_50%_, and D_2%_ of the Prior region are 22.4 Gy, 24.5 Gy, and 26.1 Gy, respectively GTV: gross tumor volume; DVHs: dose-volume histograms; CE: contrast-enhanced; WIs: weighted images; VMA: volumetric-modulated arc; WBRT: whole-brain radiotherapy; BED_10_: a biologically effective dose based on the linear-quadratic formula with an alpha/beta ratio of 10; D_X%_: a minimum dose encompassing at least X% of a target volume

The extra-CNS lesions except for the single PM were stable for eight years after the fifth-line of chemotherapy. However, the PM showed progression 16.9 months after discontinuation of nivolumab, for which stereotactic ablative radiotherapy (SABR), the first and last thoracic irradiation, was performed (details not described) (Figure 2). However, bilateral PMs and left pleural dissemination developed one month after SABR. Therefore, seventh-line chemotherapy, including paclitaxel and bevacizumab, was initiated, which resulted in transient stabilization of the disease for five months but led to difficulty in walking due to peripheral neurotoxicity. However, six months after the seventh line of chemotherapy (15.5 months after re-SRS), multiple new BMs (>17 lesions, images not shown) developed, and the left pleural dissemination progressed (Figure 1), for which an eighth line of gemcitabine (GEM) and salvage whole-brain radiotherapy (WBRT) were administered (Figures 2, 8).

**Figure 9:**
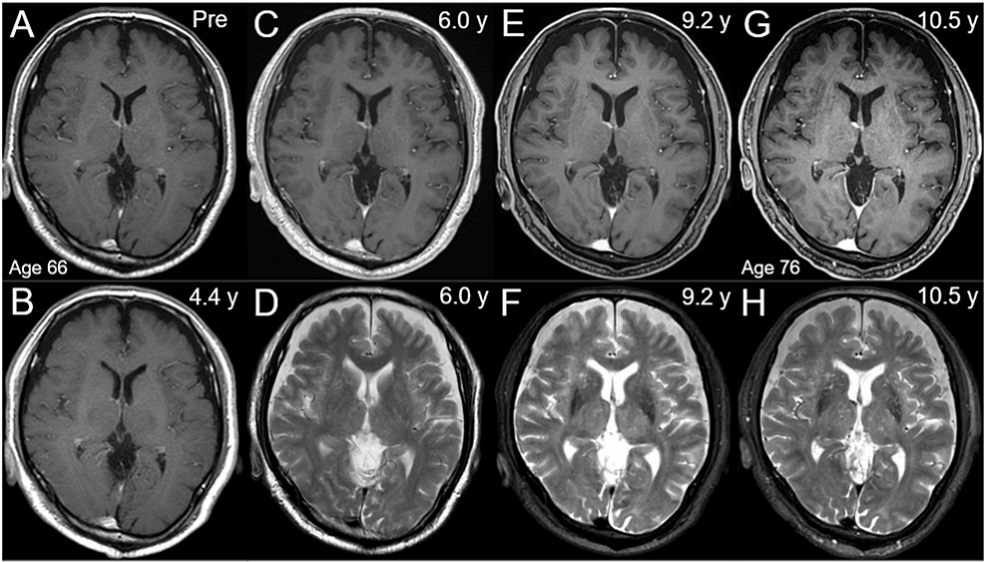
Morphological changes of the brain on the MRI before and after the initiation of anti-cancer treatment The images show axial CE-T1-WIs (A-C, E, G) and axial T2-WIs (D, F, H); before the first-line chemotherapy (Pre, at age 66) (A); at 4.4 years (y) after the first-line chemotherapy (at the time of detecting the tiny BM) (B); at 6.0 years (before the initial SRS) (C, D); at 9.2 years (before re-SRS) (E, F); and at 10.5 years (before initiating WBRT, at age 76) (G, H) (A-H) These images are shown at the same magnification and coordinates under co-registration and fusions. (A, B) T2-WIs were not acquired until six years after initiating chemotherapy. (A-H) Over the 10.5 years, the parenchymal morphology and white matter signal changes were minimal, and there was no obvious increase in bilateral subdural fluid collections MRI: magnetic resonance imaging; CE: contrast-enhanced; WI: weighted images; BM: brain metastasis; SRS: stereotactic radiosurgery; WBRT: whole brain radiotherapy

Before the WBRT, the patient was independent in terms of carrying out activities of daily living (KPS: 80%) and had no obvious cognitive decline, with inconspicuous morphological age-related changes in the brain (Figure 9).

The WBRT was commenced on the second day after initiating GEM at 80% dose (800 mg/m^2^). WBRT with 30 Gy/10 fr (D_28%_) was implemented using Agility® (Elekta AB) mounted in Infinity® (Elekta AB) with a 6 MV FF beam via a single isocenter [4]. The dose distribution was optimized with VMA to ensure simultaneously integrated boosts with 46-52 Gy to the GTV margin of 17 lesions, dose attenuation in the previously irradiated region, and homogeneous dose distribution of the other whole brain region, while sparing extra-meningeal organs, including the parotid glands and auditory apparatuses. The arc arrangement consisted of one coplanar arc and two non-coplanar arcs with each arc length of 180º, which are allocated at 60º intervals to divide the cranial hemisphere evenly. The collimator angles for each arc were separately set to be 90º, 45º, and 135º.

After completing WBRT, the patient developed anorexia, hypogeusia, and a worsened left pleural effusion. Pathological examination of the pleural effusion showed no major genetic alterations and programmed cell death ligand-1 (PD-L1) expression of <1%. GEM was administered four times; however, the left pleural effusion progressed, and the fifth dose was discontinued. Thereafter, a ninth line of atezolizumab (1200 mg/body) was administered while puncturing and draining the pleural effusion as appropriate, resulting in the progression of the left thoracic dissemination, along with a precipitous increase in CYFRA only (Figure 2). The patient complained of severe anorexia and nausea 2.3 months after completing WBRT, which persisted for two weeks. Systemic examination revealed limited gastric metastasis; however, no other causes of severe anorexia were found. Thereafter, the patient’s condition rapidly deteriorated due to a bacterial infection, eventually leading to his demise (Figure 2). The head CT images obtained on the day before the patient's death revealed no notable abnormalities (images not shown).

## Discussion

Re-SRS in the present case was performed with curative intent, as the last opportunity to eradicate the recurrent lesion, considering the possibility of long-term survival and the presumed radio-resistance of pan-negative LAC [3,9,10]. The GTV marginal dose of re-SRS in the present case seems to be one of the highest in the world: the BED_10_ of 81.6 Gy is equivalent to a single fraction of 24 Gy [3,7,9]. Matsuyama et al. have reported the significance of a BED_10_ of ≥80 Gy covering 1-2 mm outside a GTV in multi-fr SRS, to ensure excellent local control of BMs in non-small lung cancer (NSCLC) [11]; this scheme has hardly been followed up or validated since 2013, and no other facilities seem to perform SRS in accordance with this policy, especially in the re-irradiation setting [1-4]. In addition, the extremely inhomogeneous GTV dose similar to the 50-60% IDS covering of LGK was actively adopted to ensure the excellent dose concentration characteristic of SRS, along with a moderate dose spillage margin outside the GTV [8]. The steep dose increase inside the GTV boundary may be advantageous for eradicating the potentially radioresistant viable tissue concomitantly with the central necrotic region within the GTV [8,12]. The re-SRS scheme resulted in sustained regression without any local AREs for 19 months, despite the addition of WBRT. Notably, the signal changes in the surrounding brain were least noticeable 19 months after the re-SRS (Figure 7). Although the administration of bevacizumab and steroids may attenuate the AREs to some extent, the achievement of sufficient tumor necrosis would contribute to lesion stabilization. If the maximum response is only a partial one, it will eventually smolder due to regrowth unless controlled by systemic therapy [3,9,10].

One of the inherent limitations of this report is the lack of pathological verification for the local progression following the initial SRS. For the local progression, a considerable number of facilities may determine that the radiation effects are dominant and choose to observe it. Even if pseudo-progression is dominant, the clinical course after re-SRS provides important indications regarding safety. In the initial SRS, although even a single fraction could be tolerated with the GTV size, three fractions were selected, in consideration of the long-term safety [13,14]. In addition, the prescription dose was modest with the BED_10_ of 51.3 Gy, equivalent to a single fraction of 18.2 Gy (Figure 4), and therefore the GTV boundary may not have been sufficiently covered with 36.3 Gy (BED_10_ of 80 Gy) [3,4]. Nonetheless, in addition to the moderate prescription dose in the initial SRS, the relatively small GTV with the superficial location without involving the deep white matter and adequate dose fractionation in the re-SRS may have rendered the re-irradiated region immune to AREs for 19 months [7,10,13].

The 10.8-year treatment history of the present case also points to other noticeable findings. Chemotherapy- and subsequent immunotherapy-centered anti-cancer treatments can lead to more than 10 years of survival in patients harboring pan-negative LAC with systemic metastases who are unfit for targeted agents. In particular, the fifth line of CBDCA plus nab-PTX alone without consolidative thoracic irradiation resulted in a four-year sustained regression of the bulky primary lesion refractory to the four previous regimens of chemotherapy. In addition, the subsequent PD-L1 inhibitor yielded stabilization of the limited progression for more than 3.5 years (1.5 years after the discontinuation).

The present case also suggests that pseudo-progression with transient elevations of CEA and CYFRA may occur immediately after the administration of steroids following the discontinuation of nivolumab due to nivolumab-related BP. The relationship between the onset/treatment of BP and tumor markers has not yet been sufficiently investigated [15]. In addition, both CEA and CYFRA generally reflected the disease status and treatment responses, except for the elevation of CEA alone at the development of the BM and PM (Figure 2). In contrast, the final contralateral thoracic dissemination was associated with the elevation of CYFRA alone (Figure 2), which may be relevant to the intrinsic and variable histogenetic heterogeneity of LAC [16]. 

Our patient also developed persistent severe anorexia after the completion of WBRT, which warrants further investigation into the safety of concurrent use of GEM and WBRT. Although the concurrent administration of systemic chemotherapy can significantly augment the efficacy of WBRT [17], GEM can induce significant neurotoxicity when used concurrently with WBRT [18,19]. Therefore, the persistent anorexia may be attributed to the concurrent administration of GEM and WBRT. Simultaneous administration of GEM and WBRT may be better avoided hereafter.

Attempts have been made to supplement the insufficient efficacy of general SRS with reduced-dose WBRT [20]. If WBRT had been used earlier in the present case, the brain morphology and cognitive function would have been considerably different from those shown in Figure 9 (E-H). Given the variability of current SRS schemes, there is still much scope and necessity to reconsider the dose fractionation and distribution of SRS itself [3,4,8,9,12]. Focal irradiation with reservation of WBRT and an appropriate combination of systemic therapy will increasingly play a central role in the management of patients harboring limited BMs from NSCLC and even small-cell lung cancer [4,17], just as whole-organ irradiation is rarely performed for lung or liver metastases. The first step to its success is to ensure the long-term efficacy and safety of SRS, for which SRS should aim for complete local tumor eradication beyond a good partial response, especially for limited BMs, in either the first-line or re-irradiation setting [3,7,9]. In addition, flexible and appropriate dose fractionation should be used instead of sticking to a limited one [3,7,8].

## Conclusions

For local failure with a GTV of ≤1.2 cm^3^ following prior SRS for a BM from pan-negative LAC, re-SRS with 43.6 Gy/5 fr (BED_10_ of 81.6 Gy) covering the GTV and internal steep dose increase can provide excellent tumor control without AREs for 19 months. In addition to adequate consideration in prior SRS for reducing the risk of brain radionecrosis, a relatively small GTV with a superficial location and adequate dose fractionation in re-SRS may render a re-irradiated region immune to AREs after re-SRS with a high BED_10_. Long-term survival in years can be achieved by chemoimmunotherapy in patients harboring pan-negative LAC with limited systemic metastases who are unfit for targeted agents, and SRS for limited BMs in such scenarios should aim for complete local tumor eradication beyond a partial response in either the first-line or re-irradiation setting.
